# Sham-Chewing in Sows Is Associated With Decreased Fear Responses in Their Offspring

**DOI:** 10.3389/fvets.2019.00390

**Published:** 2019-11-19

**Authors:** Patricia Tatemoto, Thiago Bernardino, Luana Alves, Adroaldo José Zanella

**Affiliations:** Department of Veterinary Medicine and Animal Health, Center for Comparative Studies in Sustainability, Health and Welfare, School of Veterinary Medicine and Animal Science, FMVZ, University of São Paulo, São Paulo, Brazil

**Keywords:** cortisol, cortisone, gestation, prenatal stress, placenta, stereotypies

## Abstract

We hypothesized that sham-chewing expressed by the dam during gestation affects fetus programming. The goal of this study was to assess the effects of maternal sham-chewing on offspring welfare indicators, such as behavior and physiology. Sows that exhibited consistent sham-chewing on at least two of 6 days of observation (*N* = 7) were compared with sows that had never performed sham-chewing (non-sham-chewing sows; *N* = 4) during these 6 days. Salivary samples from sows and piglets were collected and cortisol concentrations were analyzed to assess the hypothalamic pituitary adrenal (HPA) axis activity as cortisol is a physiological indicator of welfare. Moreover, placental tissue was collected, right after farrowing, to assess cortisol and cortisone concentration. Piglet behavior and fear tests were performed after weaning (one couple per sow). In the fear tests, data was collected in an open field test to determine the states of fear indicators. Non-sham-chewing sows had lower concentrations of cortisol on days 91 and 92 of gestation in the morning. In addition to this, placental cortisol was higher among sham-chewing sows than non-sham-chewing sows. In the open field test, piglets born from non-sham-chewing sows demonstrated more latency to move in the arena and less activity, indicating more fear. Based on our data, we concluded that the expression of maternal sham-chewing is related to less fear in their offspring. Although stereotypies have been studied, attention has not been devoted to the effects of the prenatal period in considering a fetal reprogramming approach.

## Introduction

Stereotypic behaviors or “stereotypies” are a wide range of repetitive and apparently functionless patterns that often develop in environments that likely contribute to poor animal welfare (1). This behavior often develops in animals housed in environments with few stimuli, or that involve physical restraint(s), fear, and/or frustration (1). Stereotypic behaviors is also affected by a genetic component (2) and personality predisposition (3, 4). Stereotypies may also be an inherited pattern for some species, e.g., courtship. Stereotypies in general have a multifactorial cause, in which there is a synergetic effect of internal and external stimulus triggering their expression. However, some variables have a greater impact on the triggering of stereotypic behavior than others when the diverse range of environmental factors are considered (5).

On this study, we considered only the stereotypy sham-chewing. Oral stereotypies have been discussed as a beneficial strategy against gastrointestinal acidity (6). Researchers suggests that sham-chewing is related to foraging and other natural behaviors (6–8). The behavioral sequences of sham-chewing could be as a response to frustration for natural foraging (6) or just the appetitive behavior without the consummatory phase that would be the manipulation of food.

Stereotypic behavior is often considered to be an indicator of welfare (1, 9, 10) because behaviors provide clues about psychological states that are difficult to assess. Therefore, sham-chewing can be considered an indicator of animal welfare. However, stereotypies are sometimes not consistent with cortisol levels (11), a physiological welfare indicator involved in the stress cascade (12, 13). Some researchers propose, based on the “Coping Hypotheses,” that the performance of stereotypies reduces distress (14, 15).

However, it remains unclear how the long-term effects of stereotypies influence phenotypes in offspring. In mammals, pregnancy has an important role in shaping the organism as the dam's environment may influence the offspring development during gestation. This concept has emerged from the “thrifty phenotype hypothesis,” in which neurodevelopment programming induces alterations to cope with challenges in anticipating the postnatal environment (16). In other words, the prenatal environment has the potential to affect the offspring phenotype and prepare individuals for the environment that they will be inserted into in order to prepare them to better cope with challenges. The environment in which an animal is maintained during gestation may result in changes in several offspring parameters (17–22). By this mechanism, factors such as emotional reactivity, responsiveness to stressors, and cognition can be modulated by challenges in the prenatal and neonatal periods (21, 23, 24). It was showed before that some stressors, such as negative interactions with the handler (21, 22, 25) and social stress (21), can alter emotional reactivity, social behavior, and responsiveness to stressors, cognition, and memory in the offspring. Moreover, offspring of sows that experienced less hunger during gestation have exhibited reduced aggressive behavior prior to weaning (26).

Although some studies have demonstrated that stress during pregnancy or prenatal stress can generate changes that are not necessarily pathological (17), offspring exposure to an excess of glucocorticoids affect important brain structures and lead to negative effects (21, 22, 25). Glucocorticoids are important stress hormones in adult animals, but their functions are more diverse in the fetus, and their effects may be completely different depending on gestational age and the severity and duration of the exposure (27).

The placenta has a role in protecting the fetus in the prenatal period and, therefore, modulates stressful events experienced by the maternal organism and acts as a buffer (28). In mammals, 11β-hydroxysteroid dehydrogenase enzyme type 2 (11βHSD2) in the placenta forms a barrier that protects the fetus from high levels of maternal cortisol because it oxidizes the biologically active form of cortisol in cortisone (28, 29). Chronic stressful situations have the potential to inhibit the capacity to upregulate type 2 enzyme activity, and the capacity to adapt placental 11βHSD2 is significantly reduced by previous exposure to chronic stress (30), thus reducing the protective capacity of the placenta.

The effects of prenatal stress increase cortisol concentration, which can cross the placenta and affect brain structures such as the hippocampus and amygdala, and may generate changes in the offspring's emotionality (31, 32). Exploratory and fear-related behaviors such as activity and vocalizations can be quantified from novel object and open field tests (33–36). Our goal was to evaluate the consequences of a stereotypic behavior (sham-chewing) in sows during gestation in the physiological and behavioral parameters of their offspring. In this sense, if the expression of sham-chewing is helping the sows to cope with stress, it should potentially affect fetus programming. The hypothesis is that sham-chewing expressed by the mother during gestation affects fetus programming.

## Materials and Methods

### Animals and Housing Conditions

The study was carried out at the Araporanga Farm, at Jaguariaíva, Paraná, Brazil. This study was conducted according to the ethical principles of animal experimentation and under the approval of the Ethics Committee on Animal Use of the School of Veterinary Medicine and Animal Science (University of São Paulo, Brazil), protocol number 6157201114. Authors ensured that the manuscript conforms to the ARRIVE Guidelines for Reporting Animal Research.

The analysis was performed on 11 pregnant sows (TopGen Afrodite®), from a total of 30 sows, and distributed according to a body condition score in three conventional pens with a concrete floor (10 sows per pen, in which only six were considered before the inclusion criteria). The farm staff previously made the distribution according to body conditions, considering the size and weight of the sows. The sows were from the 2nd to 7th parity order (*T*-test; *p* > 0.05). We compared four sows that had never exhibited sham-chewing (non-sham-chewing sows) with seven that consistently exhibited sham-chewing (on at least 2 of 6 days of observation) and divided them into three pens with mixed treatment.

The feed was offered twice daily, at 07:00 and 11:40 a.m., and the animals had *ad libitum* access to water. Each pen was 6 m long × 3.86 m wide with a solid and slatted concrete floor area of 3.97 m in length, and the pen walls were 0.85 m high. The feeder was 5 m long and 0.37 m wide.

The piglets were weaned at 28 days of age, vaccinated (vaccines against porcine circovirus, *Streptococcus suis, Haemophilus parasuis*, and *Mycoplasma hyopneumoniae*) and transported from the Araporanga Farm in Jaguariaíva-PR (where the first stage of the experiment was performed) to the Fernando Costa Campus of the University of São Paulo in Pirassununga-SP, which involved approximately 8 h of travel. One couple of piglets per sow was randomly selected for the second part of the experiment. During the trip, each couple was placed in a transport box (73.5 cm long × 53 cm wide × 21 cm high), making it impossible for aggressive interaction between different litters, and these were lined with straw (hay).

After weaning, the piglets were housed in suspended pens, with 6 litters per pen. Each pen consisted of 12 animals, a couple from each sow. We had three pens and the animals were grouped according to their mother's group (mixed treatment). The piglets had *ad libitum* access to food and water.

### Experimental Design

To assess the effects of sham-chewing on the offspring during gestation, we studied the behavior and salivary cortisol concentration from their piglets. The behaviors evaluated included aggressiveness, nosing, and fear-related behaviors. In addition, the glucocorticoids in the placental tissue were accessed.

### Sow Behavioral Data

To collect behavioral data, an ethogram was adapted (37) and summarized in Table 1. Behavioral measures of sows were obtained by direct observation on days 88, 89, 91, 92, 106, and 107, which represent the final one-third of the gestational period. The collection periods were conducted over two consecutive days to avoid possible interference by stressful events. The behavioral assessments were performed by direct observation at 17:30. Each animal was observed three times per uninterrupted 120 s period, totaling 6 min per animal per observation time, which, in the 6 days of observation, totaled 36 min per animal. Two observers were previously standardized to avoid bias in data collection. Observations were performed using a combination of methods for behavioral measures, which started with a scan sample, followed by a focal animal with continuous observation (uninterrupted 120 s).

**Table 1:** Definition of behaviors for data collection of pregnant sows.

**Behavior**	**Definition**
Sleep	Eyes closed, lying ventrally or laterally, no ears movements
Lying ventrally	Lying with the belly facing the ground with all the limbs under the body
Lying laterally	Lying with all the members extended laterally in one side
Standing	Body supported by the four limbs
Sham-chewing	Continuous chewing without the presence of visible food in the oral cavity
Rooting the floor	Snout touches the ground followed by head movements
Licking the floor	Tongue touches the floor and is followed by movements with the head
Interacting fence or gate	Biting or nibbling the fence wire or gate
Interacting with mats	Snout or tongue touches mats followed by head movements
Bites (E)	Bite on any parts of the body (tail, vulva, ear, body)
Facing (E)	Face to face, with fixed view to the other animal
Pushing (E)	Pushing another animal using the head or the muzzle
Vocalization (E)	Sound emission emitted by the animal

*The caption E indicates behaviors that were measured (only the events and not the duration)*.

### Salivary Cortisol and Enzyme-Linked Immunosorbent Assay

Saliva collection was performed on the same days of the behavioral evaluation, on days 88, 89, 91, 92, 106, and 107 so as to correspond to the gestation length. Two samples were collected per animal, at 06:00 a.m. and 18:00 p.m., to follow the circadian rhythm of cortisol, and the effect of sham-chewing on the hypothalamic pituitary adrenal (HPA)-axis activity. The saliva was collected using hydrophilic cotton on two roller-shaped units tied to dental floss with long tips that were presented to each animal. The animal chewed the cotton until it was saturated with saliva. The first sample collected was discarded; the collection was repeated to collect only recently produced saliva. Once the second sample was collected, it was placed into a 15 mL tube. Subsequently, the tube was packed in an icebox until the end of the collection, and the sample was frozen at −20°C until processing. Thawing was performed in a box containing ice in a temperature-controlled room. After complete thawing, the sample was centrifuged for 10 min at 1,000 × g; the supernatant was aliquoted into microtubes and frozen again at −20°C until analysis. This process assisted the removal of mucins and other components that may have interfered with the analysis. For sample analysis, 50 μl of saliva was analyzed with a cortisol enzyme immunoassay [EIA—based on (38)] in duplicate for each sow, with a pool of each gestation period, without mixing the morning and afternoon collections (e.g., with samples from 88 and 89 gestation days in the morning collection). The sensitivity of the EIA was 0.2 pg/well.

### Farrowing

Sows were moved to the farrowing crates at 108 days of gestation. The deliveries were monitored and occurred in conventional farrowing crates. At birth, each piglet had its umbilical cord tied with string kept in an antiseptic solution and dipped in iodine (10%) for 5 s. The piglets were then cleaned with paper towels, assigned a number for the order of birth on their back with a stick marker, and passed through antiseptic powder to reduce body moisture. After this initial management, the piglets were placed to ingest colostrum. On the first day of life, the piglets had their teeth ground, tail docked, ears notched, and individual weight recorded. Dextran iron supplementation was administered the day after delivery.

### Placenta Collection and Glucocorticoid Analysis

The placenta was collected from four piglets per sow, in which on standardized (size and location) piece from each placenta was cut and subsequently frozen in a −20°C freezer. All placentas from each sow were macerated together to prepare a pooled sample. Once the homogenized placenta was powder-like, 0.1 g was placed in a 1.5 mL microcentrifuge tube and 200 μL of ultrapure water was added; the suspension was vortexed for 15 s. Subsequently, 20 μL was placed in another similar tube for total protein analysis [performed in triplicate for each sample, using the Bradford protocol (39)]. One milliliter of ethyl acetate was added to the tube with water and placenta, vortexed for 15 s, and centrifuged for 15 min at 4°C. The supernatant (400 μL) was transferred to a new 1.5 mL microtube; the second (duplicate) was transferred to another tube. All samples were dried overnight in a fume hood until all the liquid volume evaporated. For glucocorticoid analysis, all samples were re-suspended in the same volume using assay buffer. The analysis was performed using the same EIA protocol for salivary cortisol (see section Salivary Cortisol and Enzyme-Linked Immunosorbent Assay). The cortisone analysis used specifically first antibody and biotin.

### Piglet Behavioral Data

Throughout the experimental period, each pen was monitored by video cameras (Seco Infrared Domeball CCD Sony®, lens 3.6 mm, Case IP66) for further analysis of behaviors, such as aggressiveness and nosing. In this study, nosing was considered in any part of a pen mates' body (duration) and agonistic interaction (duration). Agonistic interactions have been characterized for pushing, head-knocking, biting, and chasing (40–42). This study considered only the behaviors made indubitable by video recording. The duration of each agonistic interaction considered started and ended with one of the cited behaviors.

### Exploratory and Fear-Related Tests

A combination of open field and novel object tests (43) was performed to assess fear behavioral indicators or the exploratory motivations of each animal. The tests were conducted in all piglets at 41 days of age. The piglets were tested individually, and we returned then to the pen immediately after the test. The combination of tests enabled a previous piglets' habituation in the arena test, in which the open field test preceded the novel object test. The animals were tested in the arena (243 × 194.5 cm), which contained demarcations on the ground forming quadrants. The duration of each test was 5 min, totaling 10 min. The piglets were gently placed in a predetermined location in the arena and they were recorded during the test period. To record the behavior, we used IP video cameras (Seco Infrared Domeball CCD Sony®, lens 3.6 mm, Case IP66). The definition of the analyzed behaviors can be found in Table 2. The latency to walk was quantified, as well as the number of central and lateral quadrants assessed, walking time (activity), freezing time, and vocalizations (events). After this test, a novel object was inserted (traffic cone) using a pulley system in the center of the pen. Subsequent behaviors were recorded for 5 min. In this test, the latency for walking, time near the object (quadrants surrounding the object), time exploring the object (near the object with the head facing the object), freezing time, and vocalization (events) were evaluated. After each animal was tested, the pen was washed with water to reduce possible chemical clues, as well as to remove feces and urine. The pen was also washed before the start of the first test of the day to standardize the entry of piglets in the arena test.

**Table 2:** Definition of behaviors for fear tests in piglets.

**Behavior**	**Definition**
Latency to walk	Duration to start to walk
Number of quadrants	Quadrants accessed in numbers
Activity	Duration of walking to any direction
Freezing time	Duration spent without any movements
Vocalizations	Number of vocalizations
Near to object	Duration in quadrants (eight) surrounding the cone

### Saliva Collection From Piglets

Similar to the sows, saliva collection in piglets aimed to assess the activity of the HPA axis in relation to cortisol. The samples were collected on days 28, 29, 35, and 36, with two samples collected individually at 06:00 a.m. and 18:00 p.m. The collection of saliva was performed using the same methodology used for the sows (see section Salivary Cortisol and Enzyme-Linked Immunosorbent Assay). As these were piglets and did not produce significant amounts of saliva, the first cotton swabs were considered, and each sample was placed into a collection tube, placed in a box with ice, and shipped to the laboratory where they were frozen at −20°C until processing. The EIA protocol followed the same performed for the sows (section Salivary Cortisol and Enzyme-Linked Immunosorbent Assay).

### Data Analysis

Data were initially tested for normality using the Shapiro-Wilk test. Statistical tests were performed using R studio software and are specified in the respective figures. Differences with *p* < 0.05 were considered to be statistically significant and tendency considered was *p* < 0.10. The non-parametric Mann-Whitney *U*-test was used in the analysis of sows, as the number of replicates was <5 in one of the two groups.

## Results

**Figure 1:**
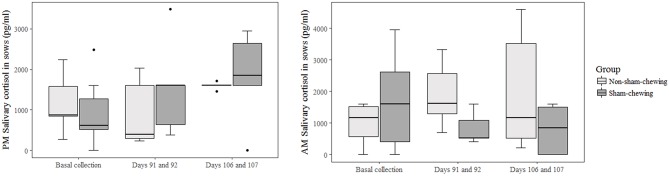
Sow salivary cortisol concentrations at 6h00 and 18h00. Using prenatal behavior observations, sows were categorized as either sham-chewing (*n* = 4; dark bars) or non-sham-chewing sows (*n* = 7; light bars). There was a difference on the morning of days 91 and 92 (Mann-Whitney *U*-test; *p* = 0.001; *Z* = 3.20) and a tendency toward greater cortisol levels on the afternoon of days 106 and 107 (Mann-Whitney *U*-test; *p* = 0.06; *Z* = 3.99).

**Figure 2:**
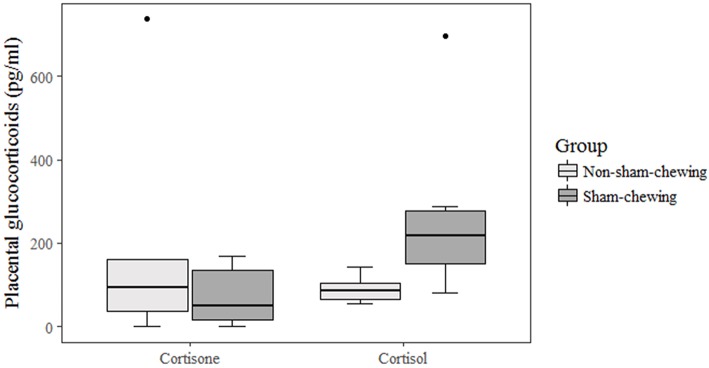
Placental glucocorticoid concentrations. Using prenatal behavior observations, sows were categorized as either sham-chewing (*n* = 4; dark bars) or non-sham-chewing sows (*n* = 7; light bars). Cortisone (Mann-Whitney *U*-test; *p* = 0.85; *Z* = 0.18) and cortisol (Mann-Whitney *U*-test; *p* = 0.04; *Z* = 2.00) concentrations in sows.

Non-sham-chewing sows had higher levels of salivary-cortisol concentrations in the morning of days 91 and 92 of gestation (Mann-Whitney *U*-test; *p* = 0.02; *Z* = 2.19; Figure 1). However, closer to delivery, sham-chewing sows had a tendency toward higher levels of salivary-cortisol on evenings 106 and 107 of gestation (Mann-Whitney *U*-test; *p* = 0.05; *Z* = −1.90; Figure 1) than non-sham-chewing sows. In additional, there were a difference in cortisol levels in the placental tissue, in which sham-chewing sows had higher levels of cortisol (Mann-Whitney *U*-test; *p* = 0.04; *Z* = 2.00; Figure 2) compared to non-sham-chewing sows. In contrast, there were no differences in the cortisone levels in the placenta between the two groups (Figure 2).

**Figure 3:**
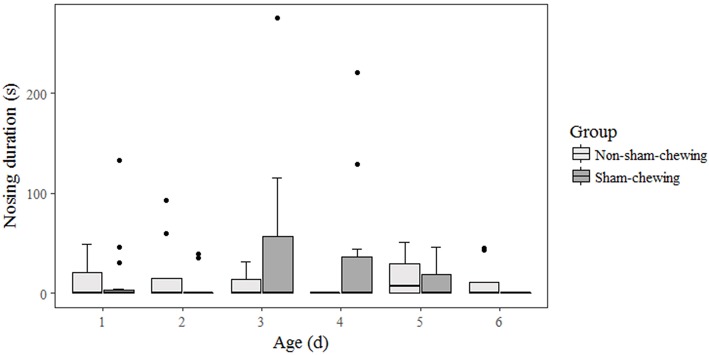
Nosing behavior in piglets. Piglets were from sows that were categorized as either sham-chewing (*n* = 4; dark bars) or non-sham-chewing sows (*n* = 7; light bars). Nosing behavior was higher on day 4 (Mann-Whitney *U*-test; *p* = 0.03; *Z* = −2.08) and lower on day 6 (Mann-Whitney *U*-test; *p* = 0.05; *Z* = 1.91) in piglets from sows that exhibited stereotypic behavior.

**Figure 4:**
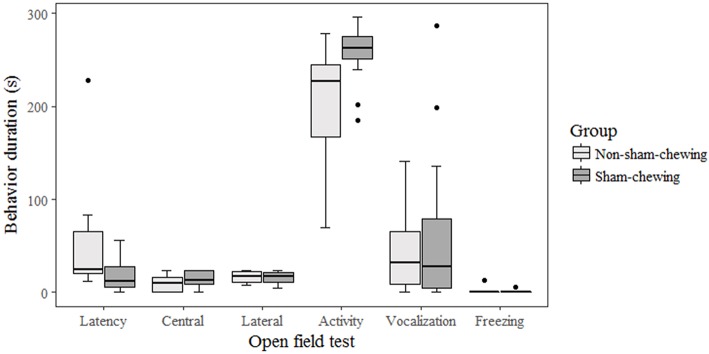
Piglet behaviors during fear and exploratory tests. Piglets were from sows that were categorized as either sham-chewing (*n* = 4; dark bars) or non-sham-chewing sows (*n* = 7; light bars). Piglets from non-sham-chewing sows demonstrated higher latency (Mann-Whitney *U*-test; *p* = 0.04; *Z* = 2.04) and less activity (Mann-Whitney *U*-test; *p* = 0.01; *Z* = −2.38). Latency was the time measured in seconds to start to walk in the arena.

Among piglets, there was no difference in salivary cortisol concentration at weaning (on the morning; *p* = 0.58; *Z* = 0.54; on the afternoon; *p* = 0.89; *Z* = 0.13) or at 35 days old (on the morning; *p* = 0.91; *Z* = −0.10; on the afternoon; *p* = 0.21; −1.22). Piglets born from sows categorized as sham-chewing did not differ from salivary cortisol (day 28 on the morning; mean = 18.42; SE = 13.42; day 28 on the afternoon; mean = 806.24; SE = 212.62; day 35 on the morning; mean = 733.47; SE = 254.98; day 35 on the afternoon; mean = 1608.94; SE = 898.37) when compared with piglets from non-sham-chewing sows (day 28 on the morning; mean = 17.00; SE = 13.01; day 28 on the afternoon; mean = 722.48; SE = 222.87; day 35 on the morning; mean = 550.93; SE = 176.67; day 35 on the afternoon; mean = 561.41; SE = 213.46). The salivary cortisol data is expressed in pg/mL. In addition, there was no difference in agonistic interactions among piglets from sham-chewing and non-sham-chewing sows (*p* > 0.05). However, in the nosing behavior, piglets from sham-chewing sows spent more time performing nosing at the age of 4 days old (Mann-Whitney *U*-test; *p* = 0.03; *Z* = −2.08) and 6 days old (Figure 3) (Mann-Whitney *U*-test; *p* = 0.05; *Z* = 1.91). During the open field and novel object tests, the differences appeared only during open field tests, in which piglets from non-sham-chewing sows showed higher latency (Mann-Whitney *U*-test; *p* = 0.04; *Z* = 2.04) and less activity (Figure 4) (Mann-Whitney *U*-test; *p* = 0.01; *Z* = −2.38). In the novel object test there was no difference among piglets from sham-chewing and non-sham-chewing sows in all measured behaviors (Mann-Whitney *U*-test, exploring the object, *p* = 0.49, *Z* = 0.68; near to object, *p* = 0.97, *Z* = 0.03; vocalizations, *p* = 0.97, *Z* = −0.03; freezing, *p* = 0.58, *Z* = −0.54).

## Discussion

Based on our data, we concluded that maternal sham-chewing expression could be related to less fear in offspring. Although stereotypies have been studied for decades, attention has not been devoted to the consequences of fetal programming and the long-term impact on future generations.

The higher cortisol concentration in non-sham-chewing sows could be associated with emotionality in the offspring outcomes, considering the potential effects of glucocorticoids on brain development (21, 22, 25). Although the association between cortisol concentration and stereotypic behavior is a controversial indicator (11), we found that non-sham-chewing sows had greater concentrations on days 91 and 92 of gestation. On days 106 and 107 of gestation, there was a tendency toward a higher concentration in sham-chewing sows. However, these measures were collected close to farrowing (predicted to occur on day 114). In this sense, when delivery is imminent, a cascade starts in offspring cortisol concentration to trigger proper physiological responses (44), and this overlaps with the stereotypy effect.

Moreover, there was a difference in the placental tissue, but only in cortisol concentration, which was higher in sham-chewing sows. As this outcome is not congruent with salivary cortisol levels, we can argue that there is difference in 11βHSD enzyme activity, which oxidizes the biologically active form of cortisol in cortisone (28, 29). Chronic stressful situations have the potential to inhibit the capacity to upregulate type 2 enzyme activity, and the capacity to adapt placental 11βHSD2 is greatly reduced by previous exposure to chronic stress (30), thus reducing the protection capacity of the placenta. Furthermore, is possible that cortisol is concentrated in the placental tissue instead of crossing and reaching the fetus' brains. In other words, the higher cortisol concentration in sham-chewing sows could result from a placenta holding this glucocorticoid and protecting the brain development in their offspring. We also have some evidence that the epigenome in the limbic system of these piglets is differentially methylated (45).

There was no difference in salivary cortisol concentration in piglets at weaning or at 35 days old. Additionally, there was no difference in aggressive behavior between piglets. However, in nosing behavior (Figure 3), piglets born of sham-chewing sows spent more time performing nosing on day 4, but not in day 6, and piglets born of non-sham-chewing sows spent more time performing nosing behavior. Nosing and belly nosing is an undesirable piglet-directed behavior expressed after weaning that can sometimes be a trigger for aggressiveness (personal observation) and cause skin lesions in the recipient when persistently performed, as is belly nosing (46). In this study, we considered nosing as any behavior directed for any part of pen mates' body. Once this motor behavior pattern precedes suckling and milk intake, it has been suggested that it may be associated with hunger or feeding (42) or even with the artificial early weaning.

In previous studies, there was a negative correlation between suckling behavior in the sow and nosing after weaning (47). Poor-quality diet post-weaning and the presence of milk did not affect the development of belly-nosing in piglets weaned between 14 and 18 days old (48). These data support the hypothesis that feeding motivation and hunger is not a causal factor of belly nosing. Moreover, there are differences between nosing (as a piglet-directed behavior) and belly nosing, for which the levels in nosing remain significantly more consistent throughout time and start the first day after weaning, instead of the peak of belly-nosing, which appears only in the second week post-weaning and starts to decrease thereafter (48). Another possibility is that the nosing we observed is a stereotypic behavior because it is consistent with the definition of repetitive behavior and appears to have no obvious function (1). As a stereotypic behavior, it can be a strategy to cope with artificial weaning in some piglets.

Moreover, it has been shown that there is a strong genetic basis for the development of stereotypies (2, 49). There is not necessarily a congruence between the type of stereotypy exhibited by mothers and offspring (2), although in this sense, it is reasonable to expect stereotypies in offspring from mothers that also performed. However, the genetic component must interact with environmental conditions to trigger this feature in the offspring. Apart from genetic predisposition, the effects of parental behavior cannot be excluded because the offspring passed the first 28 days with their mothers. Maternal behavior affects litter development, which includes behavior and emotionality (50–52).

In the fear tests, piglets from non-sham-chewing sows demonstrated higher latency and less activity, indicating more fear states. Fear is the most common emotion investigated in domestic animals (33) and this emotion is related to welfare since it is a negative emotion. However, evolutionary mechanisms shape emotions to increase fitness, and fear is a reaction to the perception of actual danger to trigger appropriate adaptive responses (33, 53). Although pigs in general have an explorative trait in their genome, it is expected that a piglet kept alone in a novel space and that is then faced with a unknown object will experience some level of fear. This response should be adaptive throughout evolution since it is a risk to be alone, exposed to predators, and away from their mates and mother. Nonetheless, it is possible to measure only the indicators of fear once, as every emotion is a subjective state (33).

We have shown in preliminary results that the expression of sham-chewing by the mother affects the offspring's emotionality (45). However, in that experiment, we considered a gradient of sows, in which we divided 28 sows in two groups, from low to high expression in terms of duration. In the open field test, piglets from sows with a high rate of stereotypies walked more in central quadrants and lateral quadrants than piglets from sows with a low rate of stereotypies. Moreover, in the novel object test the offspring from low stereotypy sows vocalized more (45). We demonstrated for the first time that stereotypic behavior expressed by the mother during gestation changes the phenotype of the offspring, in particular their emotionality (45). This outcome can be related to the stress response or reactivity, reflecting stereotypies as an indicator of welfare. In contrast, the present study adopted a focused strategy, in which we considered consistent sham-chewing throughout the days of observation. This approach enabled us to select a desirable profile for answering our question regarding the “thrifty hypothesis.” Our data indicate that it is not simply a difference in piglet emotionality, but that piglets from sows that do not exhibit stereotypic behavior exhibit more fear. To our knowledge, these results are the first to indicate that sows exhibiting sham-chewing bear piglets with less fear.

Overall, we consider that it is reasonable to accept that stereotypies are a welfare indicator. When animals are expressing stereotypies, it may indicate that the environment and context in which they are kept is not meeting their needs. However, it is possible that the individuals that are not expressing stereotypies, under the same difficult situation, are experiencing more compromised welfare, as we have shown in our studies investigating the consequences to the offspring.

## Data Availability Statement

The raw data supporting the conclusions of this manuscript will be made available by the authors, without undue reservation, to any qualified researcher.

## Ethics Statement

This study was approved by the Ethics Committee on Animal Use of the Faculty of Veterinary Medicine and Animal Science, University of São Paulo (CEUA/FMVZ, protocol number 6157201114).

## Author Contributions

PT and AZ conceptualized the study and its methodology. PT performed the formal analysis, the project administration, and wrote the original draft. AZ performed the funding acquisition, collected resources, and supervised the study. PT, TB, and LA performed the investigation. PT, TB, LA, and AZ wrote, reviewed, and edited the manuscript.

### Conflict of Interest

The authors declare that the research was conducted in the absence of any commercial or financial relationships that could be construed as a potential conflict of interest.
